# STAT3‐induced upregulation of lncRNA ABHD11‐AS1 promotes tumour progression in papillary thyroid carcinoma by regulating miR‐1301‐3p/STAT3 axis and PI3K/AKT signalling pathway

**DOI:** 10.1111/cpr.12569

**Published:** 2019-01-18

**Authors:** Juyi Wen, Hongwei Wang, Tingjun Dong, Panpan Gan, Henghu Fang, Sudong Wu, Jingjiao Li, Yuanyuan Zhang, Rui Du, Qi Zhu

**Affiliations:** ^1^ Department of Radiation and Oncology Navy General Hospital Beijing China; ^2^ Department of Neurosurgery Navy General Hospital Beijing China; ^3^ TCM‐Integrated Cancer Center of Southern Medical University GuangZhou Guangdong China; ^4^ AnHui Medical University HeFei Anhui China

**Keywords:** lncRNA ABHD11‐AS1, miR‐1301‐3p, papillary thyroid carcinoma, PI3K/AKT signalling pathway, STAT3

## Abstract

**Objectives:**

Emerging evidences indicated the importance of long non‐coding RNAs (lncRNAs) in the tumorigenesis and deterioration of malignant tumours. To our knowledge, the study about lncRNAs in papillary thyroid carcinoma (PTC) is still inadequate. ABHD11‐AS1 was highly expressed in the PTC samples of The Cancer Genome Atlas database. This study focused on the biological function and mechanism of lncRNA ABHD11‐AS1 in PTC.

**Materials and methods:**

qRT‐PCR analysis was used to examine the expression of ABHD11‐AS1 in PTC tissues and cell lines. The prognostic significance of ABHD11‐AS1 for the patients with PTC was analysed with Kaplan‐Meier analysis. The effects of ABHD11‐AS1 knockdown on the cell proliferation and metastasis were evaluated by in vitro functional assays and in vivo experiments. The molecular mechanism which contributed to the oncogenic role of ABHD11‐AS1 in PTC was explored by conducting mechanism experiments. Rescue assays were carried out for final demonstration.

**Results:**

High expression of ABHD11‐AS1 predicted poor prognosis for patients with PTC and promoted cell proliferation and metastasis in vitro and in vivo. ABHD11‐AS1 was activated by the transcription factor STAT3. ABHD11‐AS1 positively regulated PI3K/AKT signalling pathway. ABHD11‐AS1 acted as a competitive endogenous (ce) RNA to upregulate STAT3 by sponging miR‐1301‐3p.

**Conclusions:**

STAT3‐induced lncRNA ABHD11‐AS1 promoted PTC progression by regulating PI3K/AKT signalling pathway and miR‐1301‐3p/STAT3 axis.

## 1 INTRODUCTION

Thyroid cancer is a kind of commonest endocrine system malignant tumours. The mortality of patients with thyroid cancer is rapidly increasing over the past decades.^1^ Papillary thyroid carcinoma (PTC) is the commonest subtype of thyroid cancer.^2^ There are some risk factors for PTC, such as ionizing radiation exposure, thyroid nodular disease and the family history.^3^ However, the specific mechanism underlying PTC is widely elusive. Therefore, investigating the potential molecular mechanism involved in PTC progression is very essential to find novel biomarkers or therapeutic targets for PTC.

As a subtype of non‐coding RNAs (ncRNAs), long non‐coding RNAs (lncRNAs) are originally defined as transcriptional noise.^4^, ^5^ Importantly, the dysregulation of lncRNAs can affect the progression of various human diseases, including cancers. The most significant function of abnormally expressed lncRNAs is to regulate cellular processes in human cancers and determine the outcomes for cancer patients.^6^, ^7^ Epigenetic regulation is a major reason for aberrant expression of lncRNAs. For instance, H3K27 acetylation‐mediated activation induces high expression of lncRNA CCAT1 in oesophageal squamous cell carcinoma,^8^ whereas the downregulation of p53‐induced lncRNA TP53TG1 is caused by the high methylation of its gene promoter.^9^ Additionally, upregulation of lncRNAs induced by its upstream transcription factor can promote tumour progression in various human cancers.^10^, ^11^, ^12^, ^13^ The dysregulation of lncRNAs has been identified as an oncogenic factor for the tumour progression.^14^, ^15^, ^16^, ^17^ Therefore, it is crucial to investigate the mechanism of lncRNAs in human cancers.

Based on the data of The Cancer Genome Atlas (TCGA) database, ABHD11‐AS1 was significantly highly expressed in PTC samples. qRT‐PCR exanimation further detects the expression of ABHD11‐AS1 in 82 PTC tissues. Kaplan‐Meier method was to identify the effect of ABHD11‐AS1 expression on the prognosis of PTC patients. The oncogenic role of ABHD11‐AS1 in PTC was identified by performing loss‐of‐function assays. Therefore, we further detected the molecular mechanism of ABHD11‐AS1 in PTC progression. As a transcription activator of lncRNAs, STAT3 has been reported in various human cancers.^18^, ^19^ In this study, STAT3 was demonstrated to be able to activate ABHD11‐AS1 transcription. Furthermore, we applied microarray analysis and KEGG analysis to find the molecular mechanism involved in ABHD11‐AS1–mediated biological behaviours of PTC cells. Previous reports showed that STAT3 can bind to miRNAs by competing with lncRNAs.^20^, ^21^, ^22^, ^23^ Therefore, we hypothesized that ABHD11‐AS1 might positively regulate STAT3 by competitively binding to a miRNA. Bioinformatics analysis and mechanism experiments were conducted to demonstrate our hypothesis. Finally, rescue assays demonstrated the function of ABHD11‐AS1‐miR‐1301‐3p‐STAT3 axis in PTC progression.

## 2 MATERIALS AND METHODS

### 2.1 Tissue samples

Paired tissues (tumour and non‐tumour) were obtained from 82 PTC patients who received surgery at Navy General Hospital. None of these patients received chemotherapy or radiotherapy before this study. Patients with other tumours or tumour history were excluded from this study. Total samples were dissected immediately, placed on ice, snap‐frozen with liquid nitrogen and stored at −80°C for later use. This study had been approved by the ethics committee of Navy General Hospital. All patients have signed the informed consents before the study.

### 2.2 Cell culture

The BCPAP (PTC cell line) was purchased from DSMZ (Braunschweig, Germany). Nthy‐ori 3‐1 (the normal cell line) and K1 (PTC cell line) were obtained from the European Collection of Authenticated Cell Culture (ECACC, Salisbury, UK). BHP5‐16 and BHP2‐7 (PTC cell lines) were obtained from the American Type Culture Collection (ATCC; Manassas, VA, USA). RPMI 1640 (Invitrogen, Carlsbad, CA, USA) with 10% foetal bovine serum (Gibco BRL, Rockville, MD, USA), 100 mg/mL streptomycin (Invitrogen) as well as 100 U/mL penicillin was used for cell culture. Cell culture was performed at 37°C in a humidified atmosphere with 5% CO_2_.

### 2.3 Detection of mycoplasma contamination

The mycoplasma contamination of cells was detected with Myco‐PCR‐Mix mycophenolate mycoplasma detection kit (Qiao Xinzhou Bioengineering Co. Ltd., Shanghai, China). The experimental results are shown in Figure S1.

### 2.4 Cell transfection

shRNA targeted to ABHD11‐AS1 (sh‐ABHD11‐AS1) and corresponding negative control (sh‐NC) were used for ABHD11‐AS1 knockdown. The shRNA sequences were shown as follows: sh‐ABHD11‐AS1: GAGGGACCTCCAGGGACGAGAGGACAGG, sh‐NC: CGATTGTTCTTCTTTCTAGTTAAGAGAA. miR‐1301‐3p was overexpressed or silenced with miR‐1301‐3p mimics or miR‐1301‐3p inhibitors. To overexpress STAT3, the whole sequence of STAT3 was subcloned into pcDNA3.1 vector. All these plasmids and expression vectors were synthesized chemically by Shanghai Integrated Biotech Solutions Co., Ltd. (Shanghai, China). All transfections were performed using Lipofectamine 2000 (Invitrogen). Cells were collected for further analysis at 48 hours after transfection.

### 2.5 RNA extraction and qRT‐PCR

Total RNA extraction was carried out with a TRIzol reagent kit (Life Technologies, Inc, Rockville, MD, USA) in accordance with the manufacturer's instructions. RNA was reversely transcribed to complementary DNA (cDNA) using a reverse transcription kit (Thermo Fisher Scientific, Waltham, MA, USA). qRT‐PCRs were conducted with EvaGreen 2×qPCR (abm) using LightCycler^®^ 96 (Roche, Beijing, China). The thermal cycling was performed under the conditions as below: 95°C for 5 minutes, 40 cycles of 95°C for 30 seconds, 60°C for 30  seconds and 72°C for 1 minute. GAPDH and U6 were used as two internal controls. The relative RNA expression level was evaluated using the $2^{-\Delta \Delta C_{\text{T}}}$ method. The primers for PCR were shown as follows: ABHD11‐AS1 (forward): 5′‐TCCAGACAAGACTTGGTCGC‐3′, ABHD11‐AS1 (reverse): 5′‐CAGCTGGTTGTGTGGCTTTC‐3′; miR‐1301‐3p (forward): 5′‐TTACAGCTGCCTGAGAGTGACTTA‐3′, miR‐1301‐3p (reverse): 5′‐CTCTACAGCTATATTGCCAGCCA‐3′; STAT3 (forward): 5′‐CTCTTCGGGATGACAGGAGC‐3′, STAT3 (reverse): 5′‐CTTGGGCGACGGTTTGAATC‐3′; GAPDH (forward): 5′‐AACAGGAGGTCCCTACTCCC‐3′, GAPDH (reverse): 5′‐GCCATTTTGCGGTGGAAATG‐3′; U6 (forward): 5′‐GCAGACCGTTCGTCAACCTA‐3′, U6 (reverse): 5′‐AATTCTGTTTGCGGTGCGTC‐3′.

### 2.6 MTT

For cell proliferation assay, BCPAP and K1 cells were seeded onto 96‐well plates (10^4^ cells/plate) in RPMI 1640 containing 10% FBS for 24 hours. Each well was added with 0.5 g/L MTT at different time points (12, 24, 48 and 72 hours). The incubation was conducted at 37°C. The MTT crystals were solubilized using dimethylsulphoxide (DMSO; Sigma‐Aldrich and Merck KGaA, Darmstadt, Germany). The cell viability was determined by measuring the absorbance at 490 nm with a microplate reader (Tecan Infinite M200; Tecan Group Ltd., Männedorf, Switzerland).

### 2.7 Colony formation assay

Following transfection for 72 hours, total cells were trypsinized (Solarbio, Beijing, China), plated into six‐well plates at 200 cells each well and cultured for 21 days using RPMI 1640 (Invitrogen) containing 10% foetal bovine serum (Gibco) under routine conditions. Medium was replaced when needed. After that, colonies were fixed by methanol and stained by 0.5% crystal violet (Sigma). Visible colonies were counted manually.

### 2.8 Flow cytometry analysis of apoptosis

After being transfected for approximately 48 hours, BCPAP and K1 cells were harvested through trypsinization. Later, the cells were re‐suspended with PBS and the concentration of cells was adjusted to 1  × 10^6^ cells/mL. Following double staining with propidium iodide and Annexin V‐fluorescein isothiocyanate, cell apoptosis was evaluated with flow cytometry (BD Biosciences, San Jose, CA, USA).

### 2.9 Caspase‐3 activity assay

The apoptosis condition was examined by using caspase‐3 activity kit (Beyotime Institute of Biotechnology, Nantong, China). In short, after cell transfection, 2 × 10^6^ cells seeded in six‐well plates were lysed in lysis buffer (as provided in the kit) for 15 minutes at 4°C, centrifuged at 600 *g* for 15 minutes at 4°C, and the consequent cell lysates were examined for protein density applying bicinchoninic assay (Beyotime Institute of Biotechnology). An aliquot of 10 µL extracted proteins from the lysates was added into 96‐well plates and then mixed with 80 µL reaction buffer which was supplemented with caspase substrate (2 μmol/L). After incubation at 37°C for 4 hours, caspase‐3 activities were examined by the use of a Tecan microplate reader at an absorbance which was 405 nm.

### 2.10 Cell migration assay

Cell migration was examined with transwell assays. In short, indicated PTC cells were suspended in FBS‐free RPMI 1640 containing 1 μg/mL mitomycin C. Cells were then seeded into the upper well of a poly‐carbonate transwell filter with 24 wells (Millipore, Bedford, MA, USA). The lower well was added with RPMI 1640 containing 10% FBS. Following 48‐hour incubation, cells which were on the lower surface of filters were fixed and stained while cells which were on the upper surface were scraped off. The results were evaluated with Zeiss photomicroscope (Carl Zeiss Meditec, Dublin, CA, USA) and quantified through counting 10 random fields.

### 2.11 Tumour xenograft model

BCPAP cells stably transfected with sh‐NC and sh‐ABHD11‐AS1 were subcutaneously injected into 5‐week‐old male BALB/c nude mice at a density of 4 × 10^6^ cells per mouse. The nude mice were divided into two groups in accordance with the transfection: sh‐NC and sh‐ABHD11‐AS1 (n = 3 each group). The nude mice were obtained from the Animal Experimental Committee of Navy General Hospital. The nude mice used in this study are healthy. All these mice are with same age and similar weight. Four weeks later, the nude mice were sacrificed. Tumour volume and weight were measured and calculated every 7 days by using a formula (0.5 × length × width^2^). This study was approved by the Committee Ethics of Animal Center of the Qilu Hospital of Shandong University.

### 2.12 In vivo tumour metastasis assay

All animal studies had acquired the approval of the Animal Experimental Committee of Qilu Hospital of Shandong University. The BALB/c nude mice (male, 4‐5 weeks old) were obtained from the Experimental Animal Center of Navy General Hospital. The nude mice used in this study are healthy. All these mice are with same age and similar weight. To conduct in vivo metastasis assays, 1  ×  10^7^ cells were implanted subcutaneously into the right hind flank regions of the two different groups (n = 3 each group). Tumour growth was recorded with the digital callipers every 7 days. Sixty days later, all mice were sacrificed so as to observe the lung and liver metastasis. Finally, the metastatic tissues were photographed and detected by H&E staining under a microscope.

### 2.13 Immunofluorescence

Cells were grown on glass coverslips and fixed with 4% formaldehyde for 10 minutes followed with the permeabilization in 0.5% Triton X‐100 at room temperature for 15 minutes. Immunofluorescence analysis was conducted by the use of the antibodies including E‐cadherin, N‐cadherin (Cell Signaling Technology, Danvers, MA, USA) and Anti‐Mouse IgG Fab2 Alexa Fluor (R) 488 (CST). DAPI (4, 6‐diamidino‐2‐phenylindole) was used to stain cell nuclei. Following immunostaining, the observation of samples was carried out with a LEICA TCS SP5 Confocal Microscope.

### 2.14 Fluorescence in situ hybridization (FISH)

Fluorescence in situ hybridization Kit (GenePharma, Shanghai, China) was used to perform FISH assay. ABHD11‐AS1 was labelled with the FAM probe, and nucleus was stained with DAPI. U6 was used as the control of nuclear fraction, while 18S rRNA was used as the control of cytoplasmic fraction.

### 2.15 Luciferase reporter assay

The different fragment sequences predicted by JASPAR were synthesized and inserted into the pGL3‐basic vector (OMEGA Engineering Inc., Norwalk, CT, USA). Subsequently, the constructed vectors were transfected into BCPAP and K1 cells along with STAT3 expression vector or NC. Forty‐eight hours later, the luciferase activity was evaluated with a Dual Luciferase Assay Kit (OMEGA Engineering Inc).

### 2.16 ChIP

ChIP was conducted by the use of the ChIP assay kit following the manufacturer's instruction (17‐610; Millipore). Formaldehyde is applied to generate the DNA‐protein cross‐links for 20‐30 minutes. After that, lysate was sonicated to be broken DNA into a fragment size which was 200‐1000 bp. Following examination of DNA fragment size and concentration, the primary antibody, anti‐STAT3 and IgG, and protein A/G beads were added into the samples and then incubated at 4°C overnight. The cross‐linking was reversed through incubation for 4 hours at 65°C. The DNA was recovered using phenol/chloroform extraction. The chromatins that were immunoprecipitated were purified and analysed by qRT‐PCR.

### 2.17 RIP assay

The MagnaRIP RNA‐Binding Protein Immunoprecipitation Kit (Millipore) was applied to perform RIP assay. The cell lysates were incubated in a RIP buffer containing the magnetic beads which had been coated with Ago2 antibodies. Input and normal IgG were taken as controls. Proteins were digested with proteinase K. Then, the immunoprecipitated RNA was isolated. At last, qRT‐PCR was used to examine the purified RNAs.

### 2.18 RNA pull‐down assay

For pull‐down assay, miR‐1301‐3p without complementary sites with STAT3 was seen as internal reference (termed NC). miR‐1301‐3p, miR‐1301‐3p‐Mut and NC were labelled with biotin to generate Bio‐miR‐1301‐3p, Bio‐miR‐1301‐3p‐Mut and Bio‐NC by GenePharma Company (Shanghai, China). Then, they were transfected into BCPAP and K1 cells. Forty‐eight hours later, cells were harvested and incubated with Dynabeads M‐280 Streptavidin (Invitrogen) for 10 minutes, and washed three times with buffer solution. The enrichments of bound RNAs were quantified and analysed by qRT‐PCR.

### 2.19 Western blotting

RIPA lysis buffer (Beyotime Biotechnology) containing protease inhibitors (Roche) was applied to extract the proteins. Quantification of proteins was carried out applying the BCA™ Protein Assay Kit (Pierce, Appleton, WI, USA). After that, proteins (30 μg/sample) were loaded and electrophoresed with 10% sodium dodecyl sulphate‐polyacrylamide gel electrophoresis, followed by the transfer to the polyvinylidene difluoride membranes. The primary antibody used in this study was as below: PI3K (ab86714), p‐P13K (ab191606), AKT (ab81283), p‐AKT (ab38449), mTOR (ab32028), p‐mTOR (ab109268), E‐cadherin (ab76055), β‐catenin (ab32572), N‐cadherin (ab18203), vimentin (ab92547) and GAPDH (ab8245). The antibodies were prepared in 5% blocking buffer with a dilution of 1:1000, incubated with the membrane at 4°C for 2 hours, washed twice with PBS and later cultivated with the use of secondary antibody (1:2000) which was marked by horseradish peroxidase at room temperature for 2 hours. The antibodies used in this study were purchased from Abcam (Cambridge, MA, USA). Immobilon Western Chemiluminescent HRP Substrate (200 μL; Millipore) was used to cover the surface of membrane. Finally, the signals were captured, and the concentration of the bands was quantified using Image Lab™ software (Bio‐Rad Laboratories, Hercules, CA, USA).

### 2.20 Statistical analysis

Data of all experiments were presented as mean ± standard deviation (SD). All experimental procedures were repeated at least three times. Statistical analysis was conducted using GraphPad Prism 6.0 statistical software (GraphPad Software Inc, La Jolla, CA, USA). Student's *t* test for two groups or Bonferroni test for more than two groups was used to analyse the differences. Kaplan‐Meier method was used to analyse the overall survival of PTC patients with high or low expression of ABHD11‐AS1. Expression association was analysed with Spearman's correlation analysis. *P* value <0.05 implied a statistically significant result.

## 3 RESULTS

### 3.1 Upregulation of lncRNA ABHD11‐AS1 predicted unfavourable prognosis for patients with PTC

**Figure 1:**
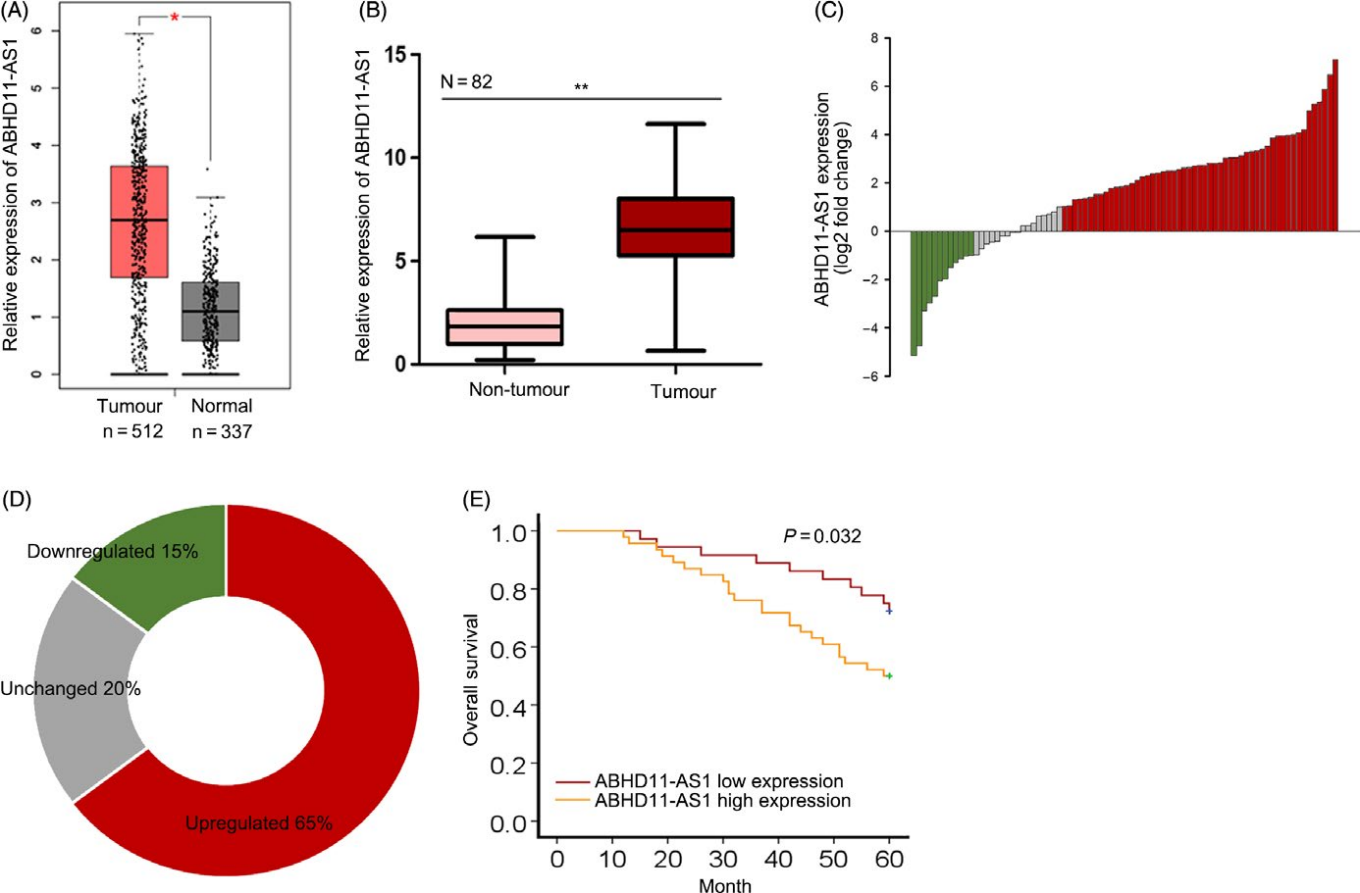
Upregulation of lncRNA ABHD11‐AS1 predicted unfavourable prognosis for patients with PTC. A, The expression of lncRNA ABHD11‐AS1 in the thyroid cancer tissues of TCGA database was detected. B, The expression of ABHD11‐AS1 was measured in 82 pairs of PTC tissues and adjacent non‐tumour tissues. C,D, The conspicuous fold change of ABHD11‐AS1 in paired tumour tissues and non‐malignant tissues was further analysed and illustrated. E, The importance of ABHD11‐AS1 expression for the prognosis of PTC patients was analysed by Kaplan‐Meier analysis. *P < 0.05, **P < 0.01. PTC: papillary thyroid carcinoma

Based on the data of TCGA database, we found that lncRNA ABHD11‐AS1 was significantly upregulated in PTC samples (Figure 1A). Next, we examined the level of ABHD11‐AS1 in 82 pairs of PTC tissues and non‐malignant tissues. Unsurprisingly, ABHD11‐AS1 was expressed higher in PCT tissues (Figure 1B). The conspicuous fold change of lncRNA ABHD11‐AS1 in paired tumour tissues and non‐malignant tissues was further analysed and illustrated (Figure 1C,D). The expression level of ABHD11 presented no significant expression difference between PTC tissue samples and normal tissue samples (Figure S2A,B). Moreover, the expression of ABHD11 was significantly changed by ABHD11‐AS1 knockdown (Figure S2C). Moreover, we found three transcripts of ABHD11 with specific sequence (Figure S2D). Then, the expression of these three transcripts was decreased by ABHD11‐AS1 knockdown (Figure S2E). Furthermore, the activity of ABHD11‐AS1 promoter was decreased in cells transfected with sh‐ABHD11‐AS1 (Figure S2F). To classify 82 PTC samples, the mean value of ABHD11‐AS1 expression was used as threshold. We found that the high expression of ABHD11‐AS1 was closely correlated with advanced TNM stage, lymph node metastasis and tumour infiltration (Table 1). Subsequently, the importance of ABHD11‐AS1 expression for the prognosis of PTC patients was analysed by Kaplan‐Meier analysis. PTC patients with higher expression of ABHD11‐AS1 had poorer overall survival than patients with lower expression of ABHD11‐AS1 (Figure 1E).

**Table 1:** Correlation between ABHD11‐AS1 expression and clinical features of PTC patients (n = 82)

Variable	ABHD11‐AS1 expression		*P*‐value
	Low	High	
Age			
<45	10	16	0.633
≥45	26	30	
Gender			
Male	23	32	0.640
Female	13	14	
Tumour size			
≤2.0	12	18	0.648
>2.0	24	28	
TNM stage			
I/II	20	11	0.006**
III/IV	16	35	
Lymph node metastasis			
Negative	20	14	0.026*
Positive	16	32	
Tumour infiltration			
Yes	22	11	0.001**
No	14	35	

Notes

Low/high by the sample median. Pearson chi‐square test.

PTC: papillary thyroid carcinoma.

* *P* < 0.05,

** *P* < 0.01 was considered to be statistically significant.

### 3.2 ABHD11‐AS1 knockdown suppressed cell proliferation and induced cell apoptosis in PTC

**Figure 2:**
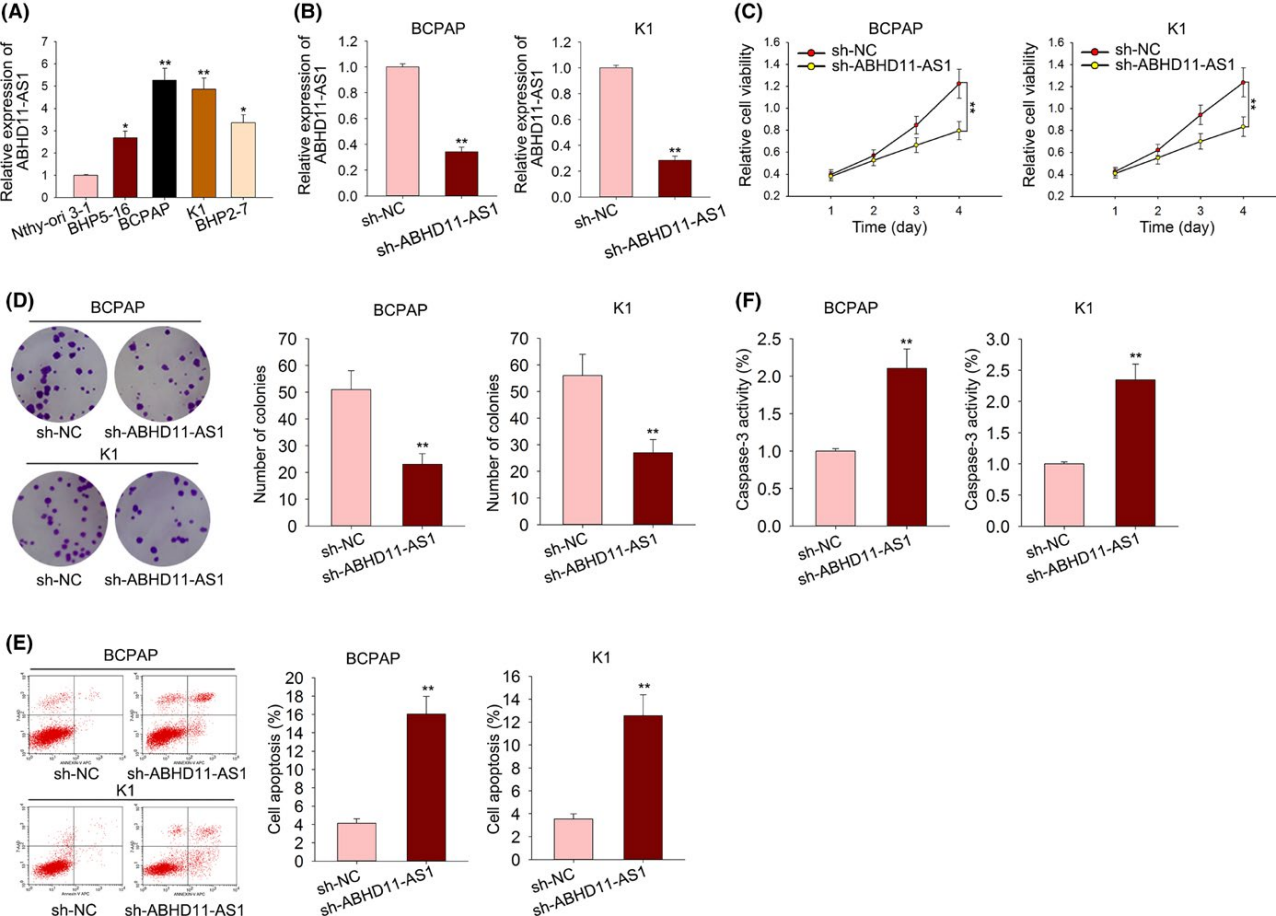
ABHD11‐AS1 knockdown suppressed cell proliferation and induced cell apoptosis in PTC. A, The expression level of ABHD11‐AS1 was examined in one normal cell line (Nthy‐ori 3‐1) and four PTC cell lines (BHP5‐16, BCPAP, K1 and BHP2‐7). B, The shRNA specifically targeted to ABHD11‐AS1 (sh‐ABHD11‐AS1) and negative control shRNA (sh‐NC) were transfected into BCPAP and K1 cells. The interference efficiency was validated by using qRT‐PCR. C,D, Cell proliferation was observed with MTT and colony formation assay after sh‐ABHD11‐AS1 transfection. E,F, Flow cytometry analysis and caspase‐3 activity detection were performed to measure the apoptotic rate of PTC cells transfected with sh‐ABHD11‐AS1. *P < 0.05, **P < 0.01. PTC: papillary thyroid carcinoma

To investigate the role of ABHD11‐AS1 in the tumour progression of PTC, functional assays were conducted in PTC cells. At first, the expression level of ABHD11‐AS1 was examined in one normal cell line (Nthy‐ori 3‐1) and four PTC cell lines (BHP5‐16, BCPAP, K1 and BHP2‐7). As expected, the higher level of ABHD11‐AS1 was detected in PTC cell lines, especially in BCPAP and K1 cells (Figure 2A). Therefore, shRNA specifically targeted to ABHD11‐AS1 (sh‐ABHD11‐AS1) and negative control shRNA (sh‐NC) were transfected into BCPAP and K1 cells to knockdown ABHD11‐AS1 effectively (Figure 2B). Subsequently, functional assays were conducted in BCPAP and K1 cells transfected with sh‐ABHD11‐AS1. Cell proliferation was found to be drastically suppressed by sh‐ABHD11‐AS1 (Figure 2C,D). To investigate whether ABHD11‐AS1 affected cell proliferation by regulating cell apoptosis, flow cytometry analysis and caspase‐3 activity detection were performed. The results manifested that cell apoptosis was promoted by silenced ABHD11‐AS1 (Figure 2E,F), indicating the positive effect of sh‐ABHD11‐AS1 on the apoptosis of PTC cells.

### 3.3 Knockdown of ABHD11‐AS1 inhibited the migration and EMT progress of PTC cells

**Figure 3:**
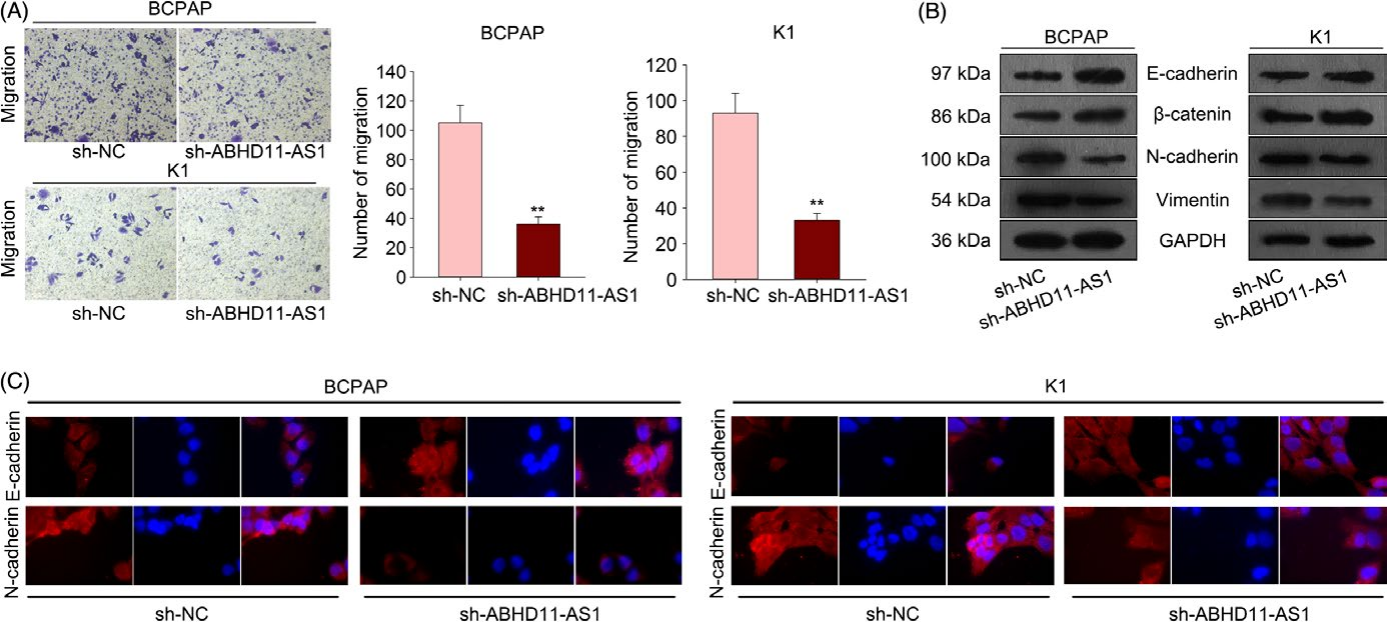
Knockdown of ABHD11‐AS1 inhibited the migration and EMT progress of PTC cells. A, The migratory capability of PTC cells transfected with sh‐ABHD11‐AS1 was tested with transwell assay. B, The influence of sh‐ABHD11‐AS1 on EMT progress was identified by detecting the protein levels of EMT markers. C, Immunofluorescence further demonstrated the reversal effect of sh‐ABHD11‐AS1 on EMT progress. **P < 0.01. PTC: papillary thyroid carcinoma

Here, we further detected the effect of differentially expressed ABHD11‐AS1 on the migration and EMT progress of PTC cell. The migratory capability of PTC cells transfected with sh‐ABHD11‐AS1 was tested with transwell assay. The result obviously showed that cell migration was noticeably suppressed by ABHD11‐AS1 knockdown (Figure 3A). Epithelial‐mesenchymal transition is known as a biological process which is closely associated with cell migration. Therefore, we further investigated the influence of sh‐ABHD11‐AS1 on EMT progress by detecting the protein levels of EMT markers. Increased level of epithelial markers (E‐cadherin, β‐catenin) and decreased level of mesenchymal markers (N‐cadherin, vimentin) indicated the reversal effect of sh‐ABHD11‐AS1 on EMT progress in PTC cells (Figure 3B). Immunofluorescence further demonstrated the reversal effect of sh‐ABHD11‐AS1 on EMT progress (Figure 3C).

### 3.4 Knockdown of ABHD11‐AS1 suppressed tumour growth and metastasis in vivo

**Figure 4:**
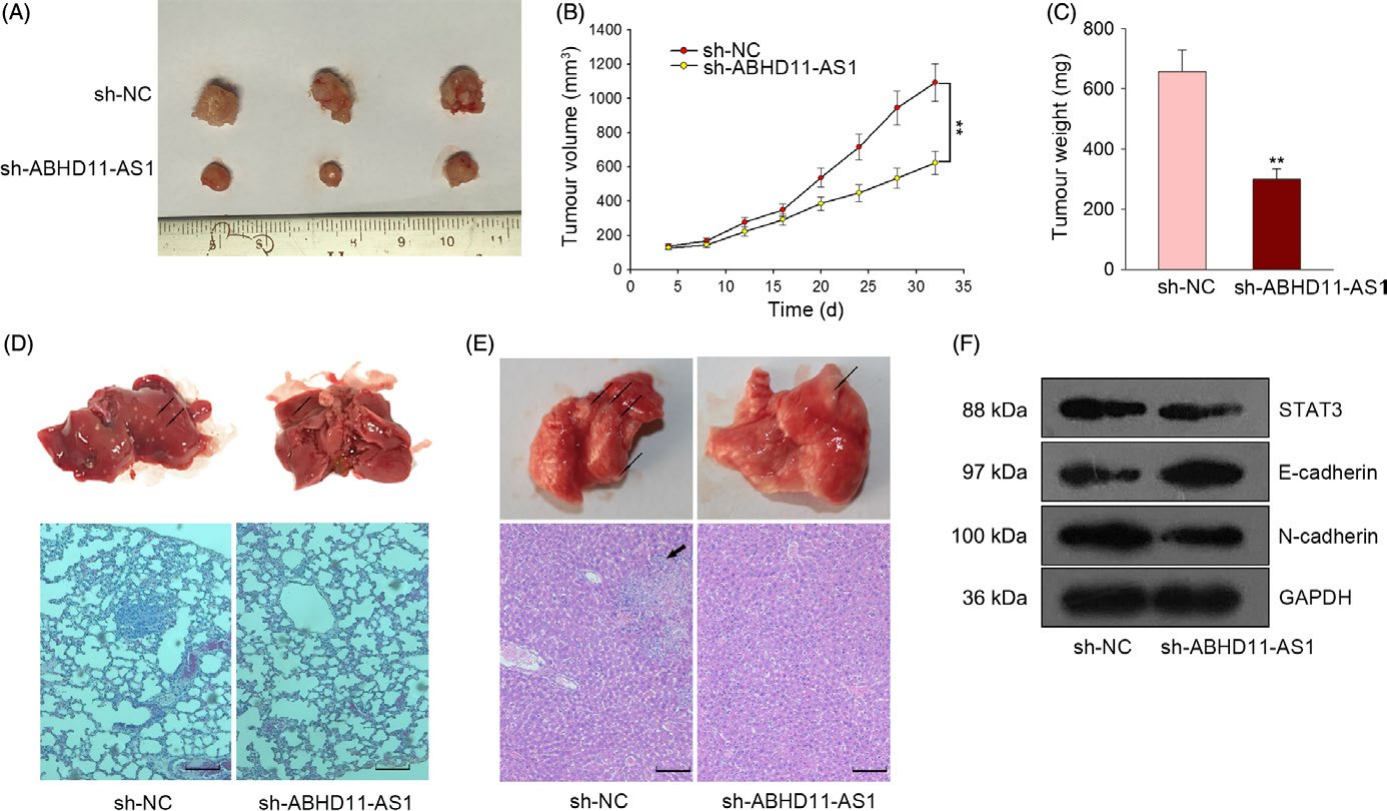
Knockdown of ABHD11‐AS1 suppressed tumour growth and metastasis in vivo. A, Tumour formation was observed in nude mice which were implanted with ABHD11‐AS1–silenced BCPAP cell. B,C. The tumour volume and tumour weight in two groups were calculated. D,E, Liver metastasis and lung metastasis were detected in response to ABHD11‐AS1 knockdown. Scale bar = 50 μm. F, The protein levels of STAT3, E‐cadherin and N‐cadherin were tested in response to ABHD11‐AS1 knockdown. **P < 0.01. PTC: papillary thyroid carcinoma

To further demonstrate the effect of ABHD11‐AS1 expression on PTC tumour progression, in vivo experiments were carried out. As shown in Figure 4A, tumour growth was drastically inhibited by silenced ABHD11‐AS1. Similarly, tumour volume and tumour weight in sh‐ABHD11‐AS1 group were smaller than sh‐NC group (Figure 4B,C). Moreover, in vivo metastasis experiment revealed that inhibition of ABHD11‐AS1 expression suppressed the liver metastasis and lung metastasis (Figure 4D,E). Then, we examined the protein levels of STAT3, E‐cadherin and N‐cadherin. The results revealed that knockdown of STAT3 led to the downregulation of STAT3, N‐cadherin, while led to the upregulation of E‐cadherin (Figure 4F). These data further indicated the oncogenic role of ABHD11‐AS1 in PTC.

### 3.5 LncRNA ABHD11‐AS1 was upregulated in PTC cells by the transcription factor STAT3

**Figure 5:**
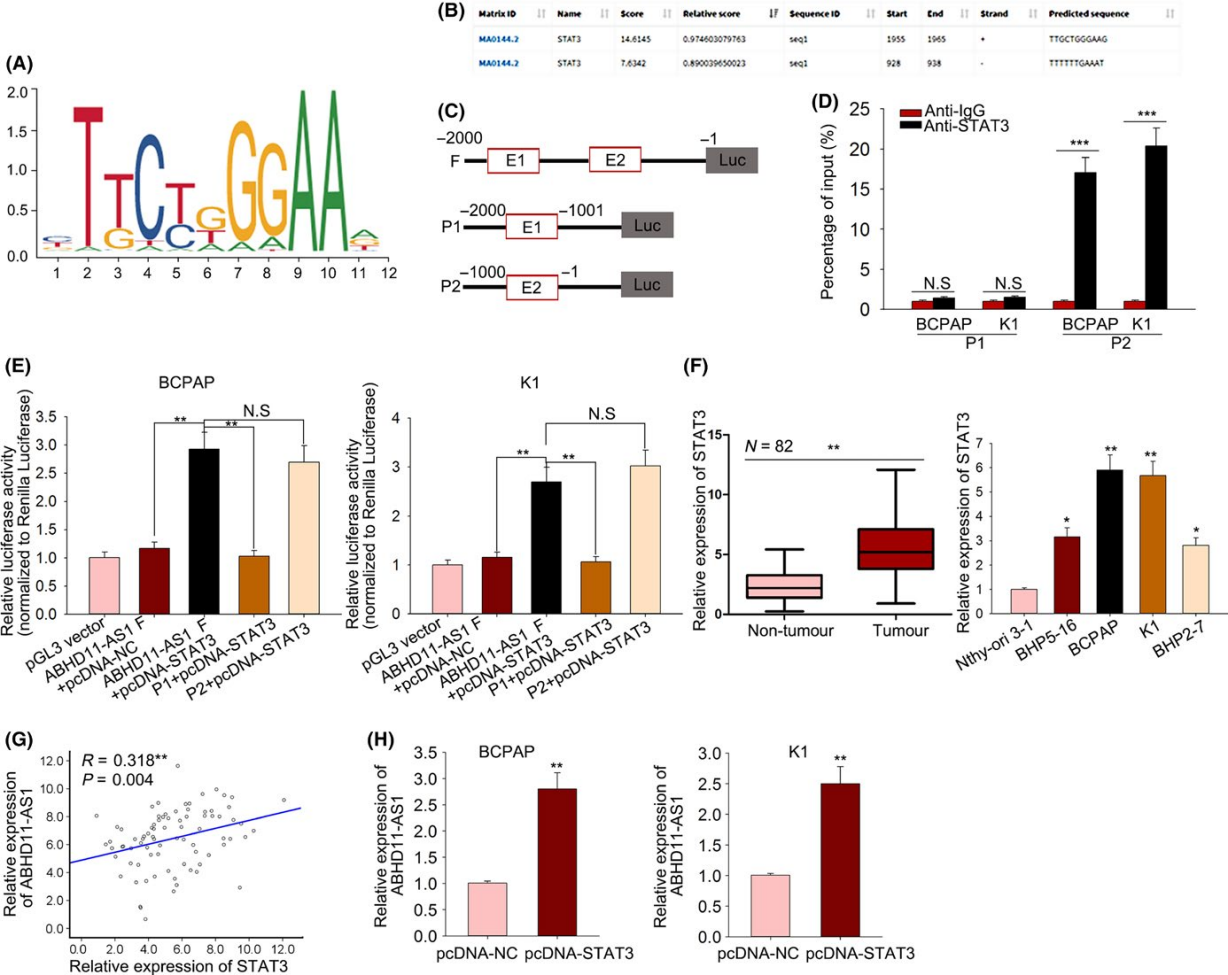
LncRNA ABHD11‐AS1 was upregulated in PTC cells by the transcription factor STAT3. A, The putative binding motif of transcription factor STAT3 was obtained from JASPAR. B,C, Top two binding sites between STAT3 and ABHD11‐AS1 promoter were chosen for further analysis. D, ChIP assay analysed the affinity of STAT3 to the part 2 (P2) of ABHD11‐AS1 promoter region. E, Luciferase activity assay further demonstrated the combination between STAT3 to the binding sequence of P2 region. F, The expression of STAT3 was detected in PTC tissues and cell lines. G, The correlation between ABHD11‐AS1 expression and STAT3 expression was then analysed in PTC tissues by Spearman's correlation analysis. H, The mRNA level of ABHD11‐AS1 was examined in PTC cells transfected with STAT3 expression vector. *P < 0.05, **P < 0.01, ***P < 0.001; N.S: no significance. LncRNA: long noncoding RNA; PTC: papillary thyroid carcinoma

All findings above suggested that upregulation of ABHD11‐AS1 is an oncogenic factor for the tumour progression of PTC. To find the reason why ABHD11‐AS1 was upregulated in PTC tissues or cell lines, mechanism investigation was performed. STAT3 was found to be an upstream transcription factor of ABHD11‐AS1. The putative binding motif of STAT3 to the ABHD11‐AS1 promoter was obtained from JASPAR (http://jaspar.genereg.net/) (Figure 5A). Top two binding sites between STAT3 and ABHD11‐AS1 promoter were chosen for further analysis (Figure 5B,C). ChIP assay validated the strong affinity of STAT3 to the part 2 (P2) of ABHD11‐AS1 promoter region (Figure 5D). Luciferase activity assay further demonstrated the combination between STAT3 to the binding sequence of P2 region (Figure 5E). All these findings indicated the binding of STAT3 to ABHD11‐AS1 promoter. To demonstrate the regulatory relationship between STAT3 and ABHD11‐AS1, the expression of STAT3 was detected in PTC tissues and cell lines. Unsurprisingly, STAT3 was definitely upregulated in PTC tissues and cell lines (Figure 5F). The positive correlation between ABHD11‐AS1 expression and STAT3 expression was then analysed in PTC tissues by Spearman's correlation analysis (Figure 5G). Furthermore, upregulation of STAT3 led to the increased expression level of ABHD11‐AS1 (Figure 5H), demonstrating the positive regulatory effect of STAT3 on the ABHD11‐AS1 expression.

### 3.6 ABHD11‐AS1 exerted oncogenic function in PTC by activating PI3K/AKT signalling pathway

**Figure 6:**
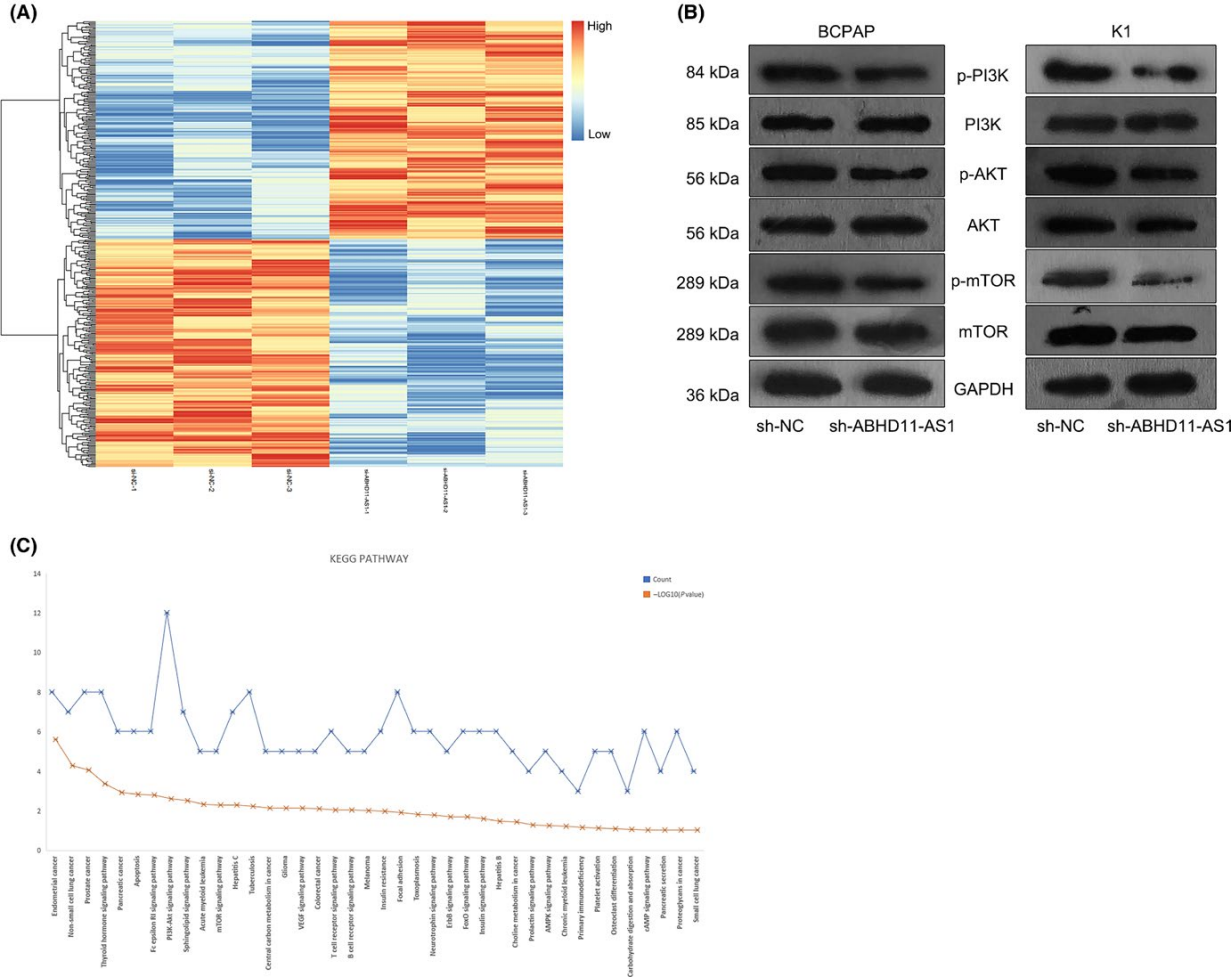
ABHD11‐AS1 exerted oncogenic function in PTC by activating PI3K/AKT signalling pathway. A, Microarray analysis was applied to detect the expression change of 500 mRNAs in ABHD11‐AS1–downregulated BCPAP cell. B, KEGG analysis analysed the related signalling pathways. C, Western blot analysis was applied to test the levels of phosphorylated PI3K, AKT and mTOR in ABHD11‐AS1–downregulated PTC cells. **P < 0.01. PTC: papillary thyroid carcinoma

To explore the downstream molecular mechanism of ABHD11‐AS1 in PTC progression, microarray analysis was applied to detect the expression change of 500 mRNAs in ABHD11‐AS1–downregulated BCPAP cell. A total of 194 mRNAs were found to be significantly upregulated, and 203 mRNAs were obviously downregulated (Figure 6A). Subsequently, KEGG analysis indicated the PI3K/AKT signalling pathway might involve in ABHD11‐AS1–mediated functions (Figure 6B). It has been reported that PI3K/AKT signalling pathway involved in lncRNAs‐mediated tumorigenesis and tumour progression. Thus, we speculated that ABHD11‐AS1 might exert its function by activating PI3K/AKT signalling pathway. Western blot analysis revealed that silenced ABHD11‐AS1 inhibited the levels of phosphorylated PI3K, AKT and mTOR (Figure 6C).

### 3.7 ABHD11‐AS1 acted as a miR‐1301‐3p sponge

**Figure 7:**
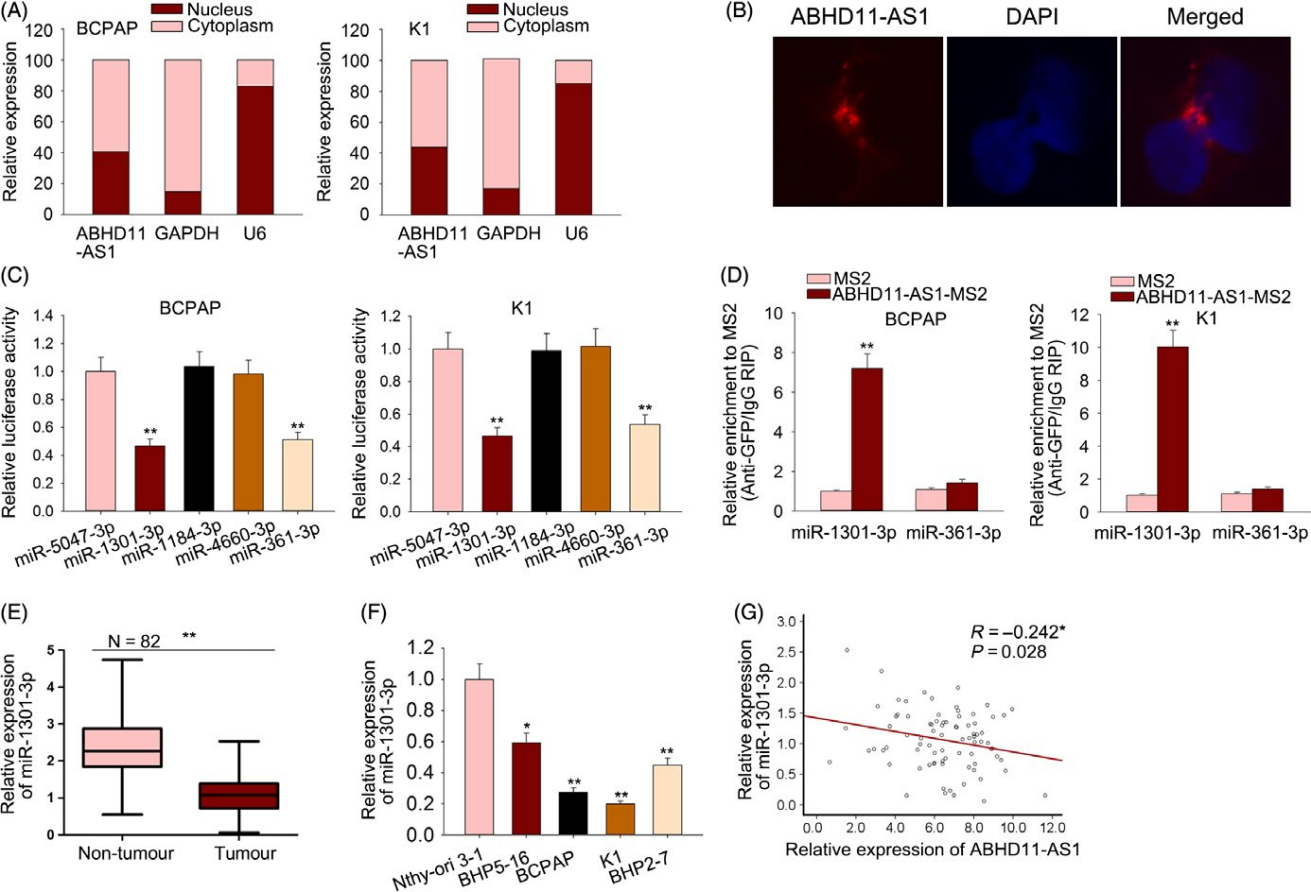
ABHD11‐AS1 acted as a miR‐1301‐3p sponge. A,B, The localization of ABHD11‐AS1 in cytoplasm or nucleus of PTC cells was identified with subcellular fractionation and FISH assay. C, The potential binding between five candidate miRNAs and ABHD11‐AS1 was assessed by luciferase reporter assay. D, RIP assay was conducted to determine the interaction between ABHD11‐AS1 and miR‐1301‐3p. E,F, The expression levels of miR‐1301‐3p in PTC tissues and cell lines. G, The expression association between ABHD11‐AS1 and miR‐1301‐3p in PTC tissues. *P < 0.05, **P < 0.01. FISH: Fluorescence in situ hybridization; PTC: papillary thyroid carcinoma

Furthermore, we investigated the downstream molecular mechanism of ABHD11‐AS1 in PTC. At first, we found that ABHD11‐AS1 expression was enriched in the cytoplasm of PTC cells (Figure 7A,B), indicating the post‐transcriptional regulation of ABHD11‐AS1. Top five miRNAs that potentially bind to ABHD11‐AS1 were found from DIANA (http://carolina.imis.athena-innovation.gr/diana_tools/web/index.php).

The binding sites between ABHD11‐AS1 and these five miRNAs are shown in Figure S3. Luciferase reporter assay suggested the binding of ABHD11‐AS1 to miR‐1301‐3p or miR‐361‐3p (Figure 7C). Furthermore, RIP assay revealed the binding of ABHD11‐AS1 to miR‐1301‐3p (Figure 7D). Next, we examined the lower expression of miR‐1301‐3p in PTC tissues and cell lines (Figure 7E,F). The negative correlation between the expression of ABHD11‐AS1 and that of miR‐1301‐3p in PTC tissues was analysed (Figure 7G).

### 3.8 ABHD11‐AS1 acted as a ceRNA by sponging miR‐1301‐3p to upregulate STAT3 expression

**Figure 8:**
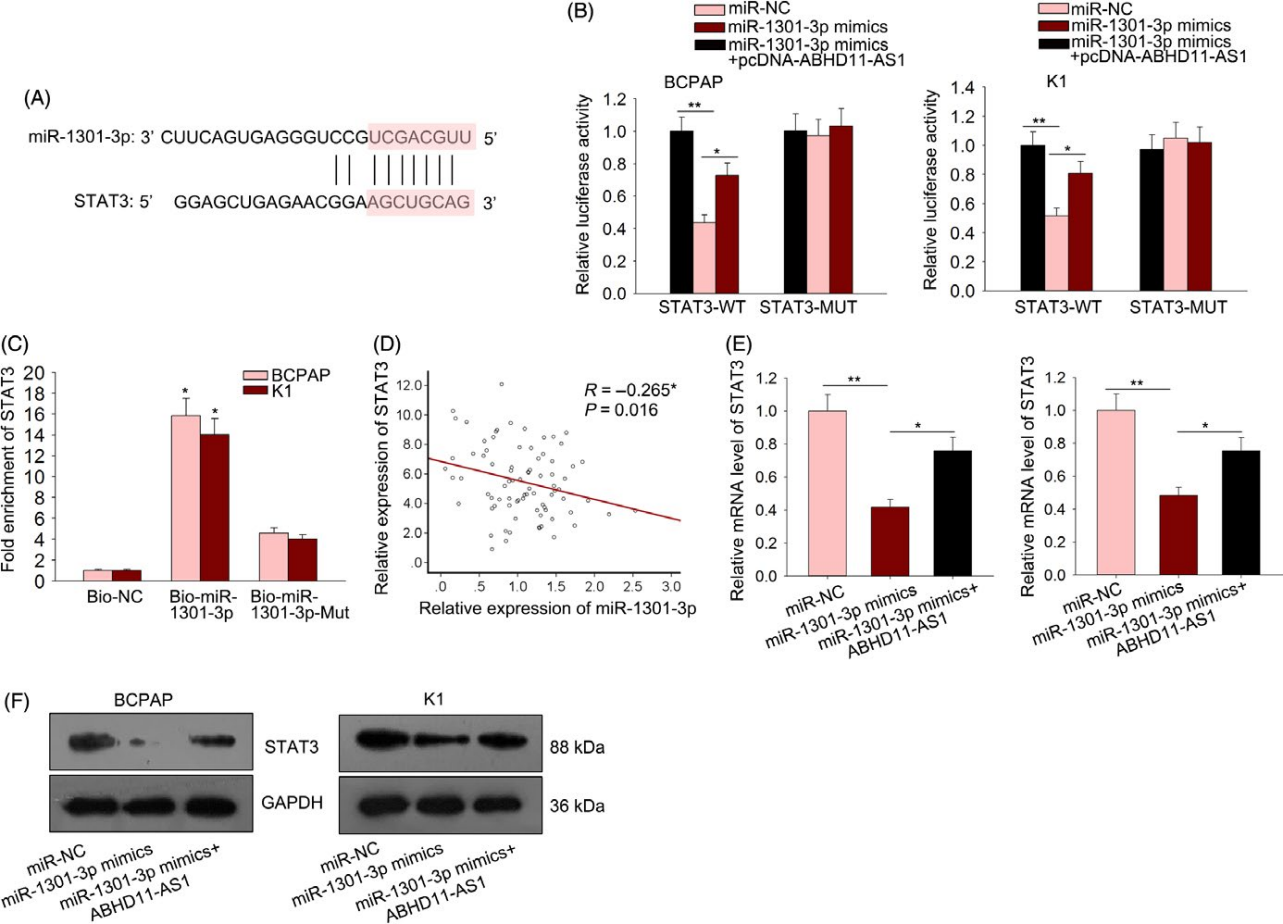
ABHD11‐AS1 acted as a ceRNA by sponging miR‐1301‐3p to upregulate STAT3 expression. A, The binding sequence between miR‐1301‐3p and STAT3 was predicted and shown. B, The luciferase activity of STAT3‐WT or STAT3‐MUT was tested in BCPAP and K1 cells transfected with miR‐1301‐3p mimics and ABHD11‐AS1 overexpression vector. C, Pull‐down assay revealed the interaction between miR‐1301‐3p and STAT3. D, The expression correlation between miR‐1301‐3p and STAT3 was analysed. E,F, The mRNA level and protein level of STAT3 were examined in BCPAP cells transfected with miR‐NC, miR‐1301‐3p mimics and ABHD11‐AS1 overexpression vector. *P < 0.05, **P < 0.01. PTC: papillary thyroid carcinoma

According to the microarray analysis, STAT3 was effectively downregulated by ABHD11‐AS1 knockdown. STAT3 has been reported in ceRNA network.^24^, ^25^, ^26^ Therefore, we investigated whether ABHD11‐AS1 positively regulated STAT3 by binding to miR‐1301‐3p, thereby forming a feedback loop. At first, the binding sequence between miR‐1301‐3p and STAT3 was predicted and shown (Figure 8A). For luciferase reporter assay, the mutant STAT3 3′UTR or wild‐type STAT3 3′UTR was co‐transfected into BCPAPA and K1 cells together with miR‐1301‐3p mimics, miR‐361‐3p mimics and negative control (miR‐NC). The results indicated that the luciferase activity of STAT3‐WT was efficiently decreased by miR‐1301‐3p mimics (Figure 8B). The effect of miR‐1301‐3p mimics on the luciferase activity of STAT3‐WT was partially attenuated by pcDNA‐ABHD11‐AS1, whereas there was no significant change on the luciferase activity of STAT3‐MUT. Furthermore, pull‐down assay revealed the interaction between miR‐1301‐3p and STAT3 (Figure 8C). The negative expression correlation between miR‐1301‐3p and STAT3 in PTC tissues was analysed (Figure 8D). Furthermore, the mRNA level and protein level of STAT3 were detected in indicated PTC cells. The expression of STAT3 decreased by miR‐1301‐3p mimics was reversed by ABHD11‐AS1 (Figure 8E,F).

### 3.9 ABHD11‐AS1‐miR‐1301‐3p‐STAT3 feedback loop promoted PTC progression

**Figure 9:**
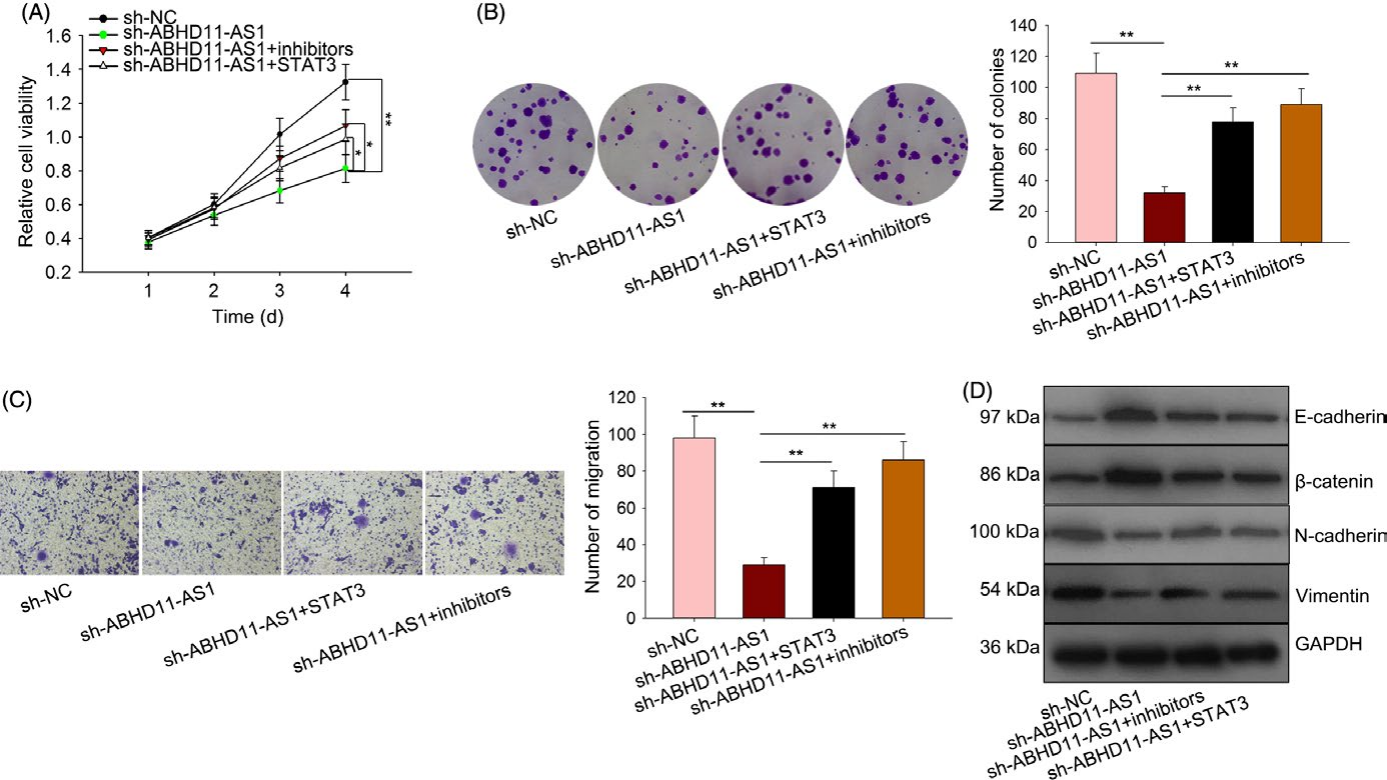
ABHD11‐AS1‐miR‐1301‐3p‐STAT3 feedback loop promoted PTC progression. A,B, The proliferative condition of BCPAP cells stably transfected with sh‐ABHD11‐AS1 was detected after co‐transfection with miR‐1301‐3p inhibitors and STAT3 expression vector. C, Transwell assay was conducted in BCPAP cell after transfection. D, EMT‐related proteins were detected in BCPAP cell after the same transfection. *P < 0.05, **P < 0.01. PTC: papillary thyroid carcinoma

To demonstrate the effect of ABHD11‐AS1‐miR‐1301‐3p‐STAT3 feedback loop on PTC progression, we designed and performed a series of rescue assays. According to the experimental results of cell proliferation assays, we found that the inhibitory effect of sh‐ABHD11‐AS1 on cell proliferation was reversed by miR‐1301‐3p inhibitors or STAT3 overexpression (Figure 9A,B). Moreover, cell migration and EMT progress suppressed by sh‐ABHD11‐AS1 were recovered by miR‐1301‐3p inhibitors or STAT3 overexpression (Figure 9C,D).

## 4 DISCUSSION

Over the several years, gene mutation has been identified to be crucial risk factor for PTC progression.^27^ However, the molecular mechanism underlying the progression of thyroid cancer is still largely unknown. Some studies have provided the evidences to show the crucial role of lncRNAs in the initiation and development of human malignant tumours,^28^, ^29^, ^30^, ^31^, ^32^ including PTC.^33^, ^34^, ^35^ This study aims to investigate the potential role of a certain lncRNA in PTC. At first, lncRNA ABHD11‐AS1 was chosen from TCGA database due to its ectopic expression in thyroid cancer samples. Subsequently, lncRNA ABHD11‐AS1 was subjected to qRT‐PCR analysis in 82 pairs of PTC tissues and corresponding non‐tumour tissues. Consistently, lncRNA ABHD11‐AS1 was conspicuously upregulated in PTC tissues. It is well known that dysregulation of lncRNAs is correlated with the prognosis of cancer patients.^36^, ^37^, ^38^, ^39^, ^40^ Here, we applied Kaplan‐Meier method to analyse the prognostic value of ABHD11‐AS1 for PTC. The PTC patients with higher expression of ABHD11‐AS1 had the poorer prognosis than those with relative lower expression of ABHD11‐AS1, indicating the potential oncogenic role of ABHD11‐AS1 in PTC. It is reported that high expression of lncRNAs contributes to tumorigenesis and tumour progression.^41^, ^42^, ^43^ To identify the oncogenic property of ABHD11‐AS1, functional assays were considered to be conducted in two different PTC cell lines. After the loss of ABHD11‐AS1 expression in these two PTC cells, cell proliferation was drastically inhibited, while cell apoptosis was accelerated and facilitated. Furthermore, cell migration and EMT progress were both reversed by silenced ABHD11‐AS1. Furthermore, in vivo experiments demonstrated the inhibitory effects of ABHD11‐AS1 knockdown on the tumour growth and metastasis. All these experimental results validated the oncogenic role of dysregulated ABHD11‐AS1 in the progression of PTC.

To our knowledge, lncRNAs exert function in tumour progression by regulating gene expression or participate in some molecular mechanism. Here, we explored the molecular mechanism involved in ABHD11‐AS1–mediated PTC progression. As we all know, transcription factors can modulate the transcription activity of lncRNAs.^44^, ^45^, ^46^ Therefore, we hypothesized that the upregulation of ABHD11‐AS1 in PTC might be caused by a certain transcription factor. Based on the search result of UCSC, STAT3 was uncovered to be the upstream transcription factor. It has been reported that STAT3 can upregulate gene expression by promoting its transcription. The binding sequence of STAT3 to the promoter region of ABHD11‐AS1 was predicted by using online bioinformatics analysis tool JASPAR. Mechanism experiments including ChIP assay and luciferase activity analysis revealed the affinity of STAT3 to ABHD11‐AS1 promoter. The relative high expression of STAT3 was detected in PTC tissues and cell lines. Naturally, the ABHD11‐AS1 expression was positively correlated with STAT3 expression in PTC tissues. The expression of ABHD11‐AS1 was positively regulated by STAT3. Hence, we confirmed that STAT3‐induced ABHD11‐AS1 promoted the progression of PTC. Furthermore, the downstream molecular mechanism of ABHD11‐AS1 was explored. Microarray analysis and KEGG analysis revealed that ABHD11‐AS1 may regulate STAT3 and PI3K/AKT signalling pathway. Western blotting analysis revealed the increased levels of p‐PI3K, p‐AKT and p‐mTOR in cells transfected with sh‐ABHD11‐AS1, indicating the activation of ABHD11‐AS1 on PI3K/AKT signalling pathway. Furthermore, we analysed the downstream molecular mechanism of ABHD11‐AS1 in PTC. Based on bioinformatics, analysis and mechanism experiments demonstrated that ABHD11‐AS1 was located in the cytoplasm of PTC cells and could bind to miR‐1301‐3p. STAT3 can be targeted by miRNAs in human cancers.^47^, ^48^, ^49^ More importantly, STAT3 can act as both transcription factor and target of lncRNA, thereby forming a feedback loop.^50^, ^51^ Therefore, we investigated whether ABHD11‐AS1 positively regulated STAT3 by sponging miR‐1301‐3p, thereby forming a feedback loop. Mechanism experiments revealed that miR‐1301‐3p could bind to STAT3 and was positively regulated by ABHD11‐AS1, indicating the ceRNA network. Finally, rescue assays validated the role ABHD11‐AS1‐miR‐1301‐3p‐STAT3 feedback loop in the progression of PTC. Our findings may help to explore the novel molecular mechanism in PTC.

## CONFLICT OF INTEREST

None.
