# Supplementary figures and images for: STAT3‐induced upregulation of lncRNA ABHD11‐AS1 promotes tumour progression in papillary thyroid carcinoma by regulating miR‐1301‐3p/STAT3 axis and PI3K/AKT signalling pathway
Source: Cell Prolif. 2019 Jan 18;52(2):e12569. doi: 10.1111/cpr.12569 (PMC6495520; doi:10.1111/cpr.12569)

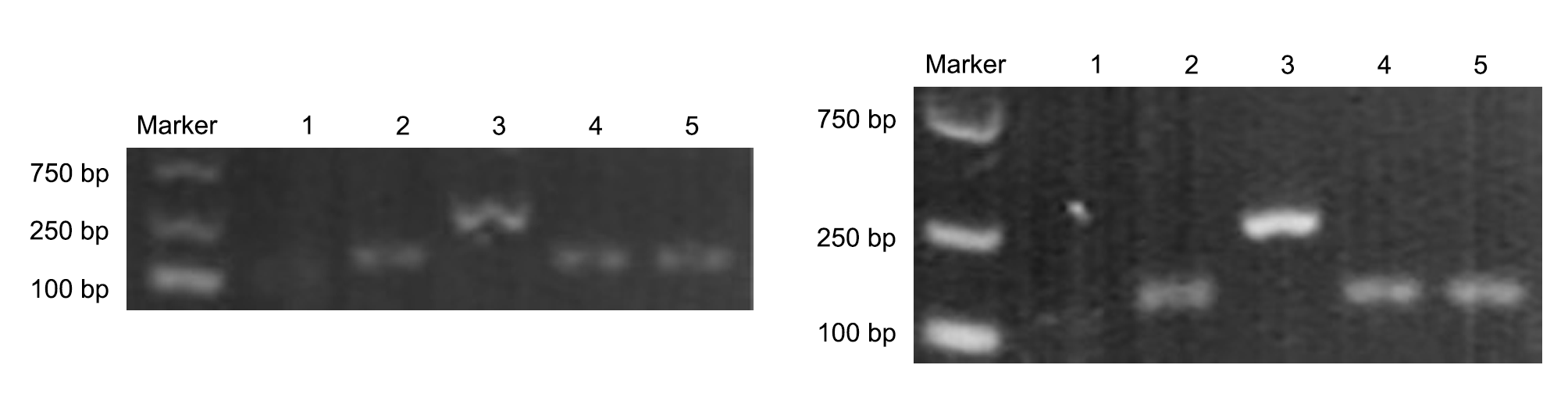

Supplement: Supplementary file 1 [file CPR-52-e12569-s001.tif]

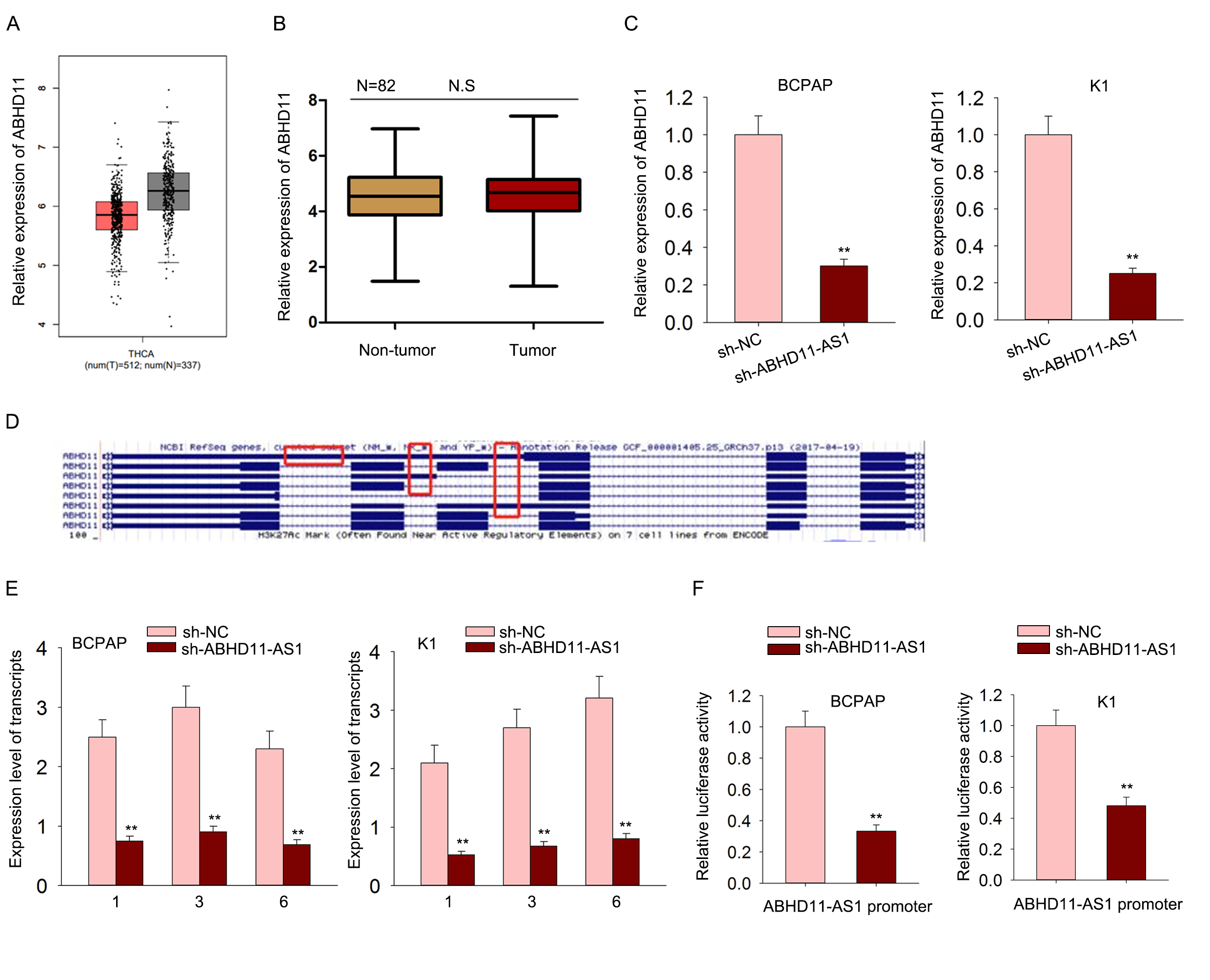

Supplement: Supplementary file 2 [file CPR-52-e12569-s002.tif]

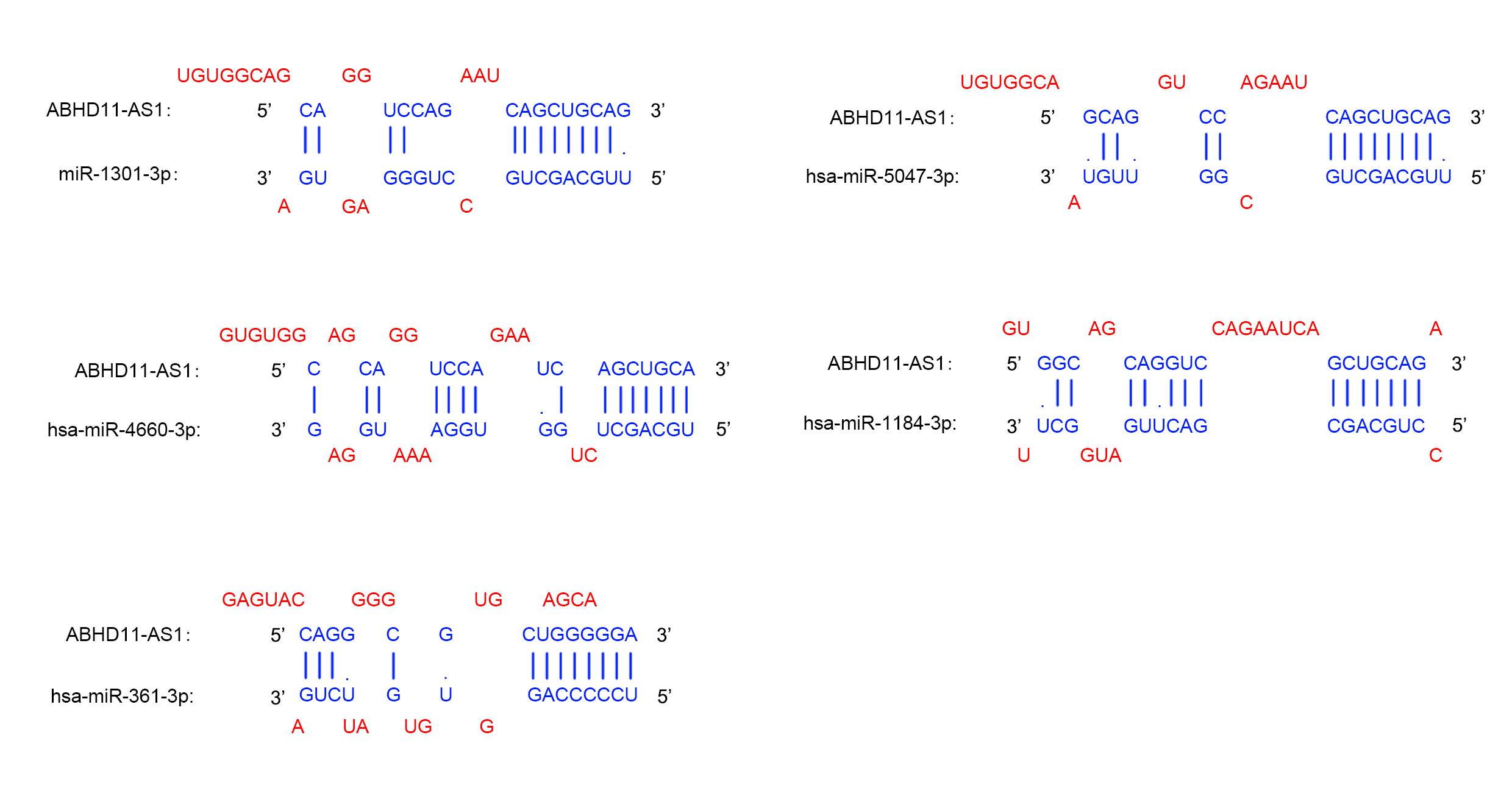

Supplement: Supplementary file 3 [file CPR-52-e12569-s003.tif]
